# Simple standing test without furosemide is useful in the diagnosis of primary aldosteronism

**DOI:** 10.1038/s41598-023-40574-y

**Published:** 2023-08-17

**Authors:** Yuichiro Iwamoto, Tomohiko Kimura, Mana Ohnishi, Takashi Kusano, Haruka Takenouchi, Hideyuki Iwamoto, Junpei Sanada, Yoshiro Fushimi, Yukino Katakura, Fuminori Tatsumi, Masashi Shimoda, Shuhei Nakanishi, Kohei Kaku, Tomoatsu Mune, Hideaki Kaneto

**Affiliations:** Division of Diabetes, Metabolism and Endocrinology, Kawasaki Medical School, 577 Matsushima, Kurashiki, 701-0192 Japan

**Keywords:** Endocrinology, Adrenal gland diseases

## Abstract

Primary aldosteronism (PA) is a well-known cause of secondary hypertension. We have long performed the simple standing test in patients with PA. On the other hand, there are few reports on the usefulness of the simple standing test in PA. This study is a single-center, retrospective, observational study. A total of 173 patients with hypertension or adrenal tumor admitted to Kawasaki Medical School were included. Eighty patients who met the exclusion criteria were excluded, and 31 patients without PA (non-PA), 26 patients with unilateral PA, and 36 patients with bilateral PA were included in the study. The simple standing test was performed after 120 min of standing or sitting followed, and the aldosterone/renin ratio (ARR) and percentage of increase plasma aldosterone concentration (%increase of PAC) was calculated. The mean ARR in the simple standing test in unilateral PA (1143 (528–2200)) and bilateral PA subjects (521 (374–765)) were significantly higher compared to non-PA subjects (152 (102–240)) (*p* < 0.0001, *p* = 0.0013, respectively). The percentage increase of PAC after standing loading was significantly lower in unilateral PA subjects (110 (96–140)) compared to non-PA subjects (187 (155–244)) (*p* = 0.0003), with no difference between non-PA and bilateral PA subjects (*p* = 0.99). The cutoff value of the ARR in the simple standing test for diagnosis of PA in this study was 364 (AUC = 0.948, sensitivity = 83.8%, specificity = 93.5%, false positive rate = 3.7%, false negative rate = 25.6%, *p* < 0.001), which was not inferior to the diagnostic performance of the captopril loading test. The diagnostic performance of the simple standing test for PA was not inferior to that of the captopril loading test. The percentage increase of PAC in unilateral PA subjects was significantly lower compared to bilateral PA subjects. These results demonstrate the usefulness of the simple standing test, which can be performed simultaneously with general screening tests of PA.

## Introduction

Primary aldosteronism (PA) is a secondary hypertension characterized by suppression of the renin-angiotensin system due to excessive aldosterone secretion^1,2^. It has been reported that PA increases cardiovascular events due to hypertension and hyperaldosteronemia^3,4^. Therefore, it is important to normalize plasma aldosterone levels as well as blood pressure in subjects with PA in order to prevent the cardiovascular events. In clinical practice, however, PA is often overlooked due to lack of measurement of plasma renin activity and aldosterone levels in subjects with hypertension. Therefore, it is recommended that we perform screening for PA especially in subjects with persistent hypertension^5,6^. The aldosterone-renin ratio (ARR) is used as a screening test for PA and can be easily evaluated by a subspecialty laboratory^7^. If screening is positive, the diagnosis is confirmed by tests such as the captopril loading test, furosemide upright test, and saline infusion test^8,9^.

Renin and aldosterone measurements vary widely depending on the time of day when the blood sample is taken, salt intake, posture, and stress^5^. At our institution, in addition to these stress tests, we empirically used plasma aldosterone concentrations after standing loading in the simple standing test as an auxiliary diagnosis^10,11^. Although the simple standing test is a very simple procedure because it does not require medication, its significance in screening and diagnosis in PA subjects is not clear. We conducted a single-center, retrospective study to determine the usefulness in PA subjects of comparing test results of the simple standing test with those of the captopril stress test, an established technique as a screening test for PA.

## Materials and methods

### Study population and patient preparation

**Figure 1:**
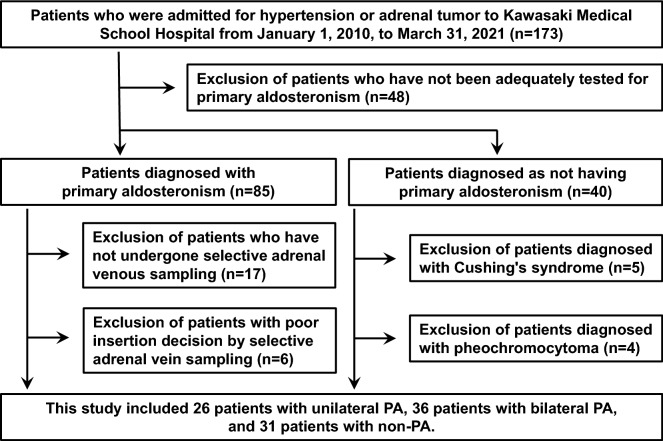
The flowchart of the participants and exclusions in this study.

This retrospective study conducted at the Division of Diabetes, Endocrinology and Metabolism, Kawasaki Medical School, included adult subjects with hypertension or adrenal tumor who were hospitalized between January 1, 2010 and March 31, 2021. The study was approved by the Institutional Review Board of Kawasaki Medical School (No. 5926–00) and was conducted in accordance with relevant guidelines and regulations. This study is a retrospective observational study using only information from medical records, and we obtained approval from the same Ethics Committee to obtain consent from patients via opt out on the Kawasaki Medical School website in accordance with domestic ethical guidelines. A flowchart of the subjects in this study is shown in Fig. 1. We first selected 173 patients who were admitted to the Division of Diabetes, Endocrinology and Metabolism, Kawasaki Medical School Hospital for close examination for hypertension or adrenal tumor from January 1, 2010 to March 31, 2021. Among them, 48 patients who had not been fully examined for PA were excluded. Based on the diagnostic criteria for PA which were described below (in the section of “Diagnosis of primary aldosteronism”), PA was diagnosed in 85 participants and the diagnosis of PA was ruled out in 40 participants. Among the participants diagnosed with PA, localization with selective adrenal vein sampling (SAVS) was possible in 61 participants, 26 with unilateral PA and 36 with bilateral PA. The results of the examination showed that PA was denied; 5 participants diagnosed with Cushing's syndrome and 4 with pheochromocytoma were excluded. Finally, 26 patients with unilateral PA, 36 patients with bilateral PA, and 31 non-PA patients were included in the study. The non-PA patients were admitted for the following diseases. Nineteen patients with essential hypertension, 10 patients with nonfunctioning adrenal adenomas, 2 patients with subclinical Cushing's syndrome, and 1 patient with adrenal calcification.

### Methods

We evaluated the usefulness of the simple standing test in participants diagnosed with unilateral and bilateral PA with SAVS and in non-PA patients. All participants in this study were evaluated for the early morning ARR and captopril loading test. Differences in clinical parameters obtained from medical records were analyzed in each group. Angiotensin-converting enzyme inhibitors, angiotensin II receptor blockers, beta-inhibitors and diuretic therapy were discontinued 2–6 weeks prior to admission. Age, sex, Brinkmann index, weight, body mass index (BMI), systolic and diastolic blood pressure, and pulse rate were calculated based on information obtained at admission. General blood tests were performed upon admission. All participants were interviewed upon admission about their pre-admission diet and their salt intake was estimated. Salt intake during hospitalization was set at 8–9 g/day. For some participants who were on strict salt restriction prior to admission, salt intake during hospitalization was set at 6 g/day. Electrolytes were measured early in the morning on the day following admission, and transtubular potassium gradient (TTKG) and fractional excretion potassium (FEK) were calculated using the following equations: TTKG = (urinary potassium concentration/serum potassium concentration)/(urine osmolality/serum osmolality), FEK = (urinary TTKG = (urinary potassium concentration/serum potassium concentration)/(urinary creatinine/serum creatinine).

### Diagnosis of primary aldosteronism

All tests were performed during hospitalization, even for participants who had previously undergone PA testing as an outpatient. Blood samples for plasma renin activity and plasma aldosterone concentration were collected upon awakening the day after admission after at least 20 min of rest and supine position and were maintained at a temperature of 2–10 °C. Plasma renin activity and plasma aldosterone concentrations were measured by the RIA method using the LumiPulse Presto (FUJIREBIO Inc., Tokyo, Japan). ARR was calculated by the following formula: (plasma aldosterone concentration/plasma renin activity) × 100. To evaluate the diurnal variation in plasma renin activity and plasma aldosterone concentration, blood samples were taken at 14:00, 20:00, and 23:00 after at least 20 min of bed rest, respectively.

The tests used to diagnose PA were either the captopril loading test or the saline loading test. For the captopril loading test, patients were placed in a resting supine position for at least 20 min upon waking, followed by 50 mg of captopril, an angiotensin-converting enzyme inhibitor, and blood was drawn 60 min later. The saline loading test was performed as follows: Patients were placed in a resting supine position for at least 20 min upon awakening, followed by 2 L of 0.9% saline administered intravenously over 4 h, and blood was collected. Positive criteria for the captopril loading test were an ARR of 200 or higher and a plasma aldosterone concentration of 60 pg/mL or higher in the saline loading test.

### Localization with selective adrenal vein sampling

For localization diagnosis by SAVS, we used the criteria described in the Japan Endocrine Society Clinical Practice Guideline for the Diagnosis and Management of primary Aldosteronism 2021 (http://www.j- endo.jp/uploads/files/news/20,210,823.pdf). The catheter insertion decision was based on the Selectivity Index (SI), which was calculated by adrenocorticotropic hormone (ACTH)-stimulated adrenal intravenous cortisol/inferior vena cava cortisol, and an insertion decision of 5 μg/dL or higher was considered successful. An aldosterone concentration of 14,000 pg/mL or higher was used as the criterion for hypersecretion. Lateralized ratio (LR) was used for localization diagnosis. LR was calculated by intravenous adrenal aldosterone concentration/cortisol (A/C) after ACTH loading. A unilateral lesion was determined if the unilateral A/C was 4 times greater than the contralateral A/C. All PA participants in the analysis in this study had SI ≥ 5 μg/dL, and localization was performed by LR. Twenty-five subjects had unilateral PA and 36 had bilateral PA.

### The simple standing test method

The procedure for the simple standing test is to maintain a standing or sitting position for 120 min after resting for at least 20 min. Blood samples were drawn at two time points: before the postural change and 120 min after the postural change, and blood samples were taken for measurement of plasma renin activity and plasma aldosterone concentration. All patients were monitored for blood pressure from the start of the simple upright test until 3 h after the end of the test. Patients were informed at the time of testing that testing would be terminated if their systolic blood pressure was less than 80 mmHg or greater than 200 mmHg or if any other adverse event occurred. No test-related adverse events occurred in this study, and all patients completed the simple standing test.

### Statistical analysis

Data are expressed as mean and standard deviation. Plasma renin activity, plasma aldosterone concentration, ARR, and %increase of PAC are shown in Median (interquartile range) because of nonparametric distribution. The primary endpoint was to evaluate the diagnostic performance of the simple standing test in subjects with possible primary aldosteronism. ANOVA was used to compare the diurnal variation of clinical parameters, plasma renin activity and plasma aldosterone concentration among the three groups, and the Tukey method was used as the post hoc test. Chi-square test was used to assess gender and antihypertensive medication adherence. To evaluate the usefulness of ARR in the early morning, ARR in the simple standing test, and ARR in the captopril loading test for the diagnosis of primary aldosteronism, ROC curves were developed for patients diagnosed with PA and non-PA, and test sensitivity and specificity were calculated, respectively. In addition, ROC curves were developed for PA subjects to calculate the cutoff value of %increase of PAC for localization diagnosis. Nominal logistic regression analysis was performed to evaluate predictive factors for localization diagnosis of PA, using age, gender, %increase of PAC, serum potassium, and the presence of unilateral adrenal lesions on CT as explanatory variables. JMP (version 16.0.2) was used for data analysis and Microsoft EXCEL for Mac (version 16.69.1) for tabulation.

## Results

### Clinical characteristics of this study subjects

**Figure 2:**
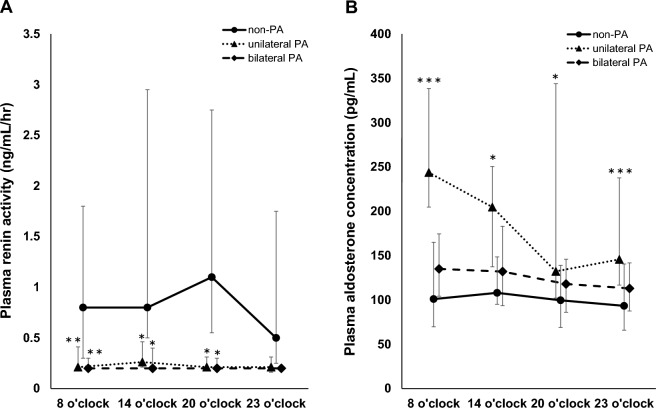
Diurnal variation of plasma renin activity (**A**). Diurnal variation of plasma aldosterone concentration (**B**). The plot represents the median and the error bars represent the interquartile range. *p < 0.05, **p < 0.005, ***p < 0.001 compared to non-PA subjects by the Tukey method.

The clinical characteristics of the subjects in this study are summarized in Table 1. The mean age of all subjects was 56 ± 11 years and BMI was 25 ± 4 kg/m^2^. There were no group differences in age, gender, BMI, blood pressure, pulse between unilateral/bilateral PA and non-PA subjects. Antihypertensive drug use rates were significantly higher in unilateral PA (73.1%) and bilateral PA (80.6%) compared to non-PA (54.8%) (*p* = 0.047). Serum sodium levels in unilateral PA subjects (143 ± 2 mmol/L) were significantly higher compared to non-PA subjects (141 ± 3 mmol/L) (*p* = 0.0059). Serum potassium and TTKG were significantly higher in unilateral PA subjects compared to non-PA subjects (*p* < 0.0001, *p* = 0.075). While serum sodium and potassium levels and TTKG of bilateral PA subjects did not differ compared to non-PA subjects. CT findings of adrenal lesions larger than 10 mm were observed in 45.2% of non-PA subjects, 96.2% of unilateral PA subjects, and 52.8% of bilateral PA subjects (*p* < 0.0001). Nine non-PA subjects had adrenal lesions with nodular lesion of the unilateral adrenal gland and five had findings of bilateral adrenal hyperplasia. All adrenal lesions in unilateral PA subjects were nodular lesions of the unilateral adrenal gland, while 68.4% of adrenal lesions in bilateral PA subjects were nodular lesions of the unilateral adrenal gland and 31.6% had findings of bilateral adrenal hyperplasia. Figure 2 shows the diurnal variation of plasma renin activity and plasma aldosterone concentration. Plasma renin activity was significantly lower in PA subjects compared to non-PA subjects (Fig. 2A). Plasma aldosterone concentrations were significantly higher in subjects with unilateral PA compared to non-PA subjects (Fig. 2B).

**Table 1:** Various clinical parameters in this study subjects.

Parameters	All subjects (n = 93)	Non-PA subjects (n = 31)	Unilateral PA subjects (n = 26)	Bilateral PA subjects (n = 36)	*P* value
Male/female	40/53	16/15	13/13	12/24	0.31
Age (years)	56 ± 11	57 ± 13	54 ± 12	58 ± 9	0.36
Body weight (kg)	65 ± 13	67 ± 11	65 ± 16	64 ± 13	0.57
BMI (kg/m^2^)	25 ± 4	25 ± 4	25 ± 5	24 ± 3	0.53
Systolic blood pressure (mmHg)	141 ± 19	141 ± 20	144 ± 15	138 ± 21	0.43
Diastolic blood pressure (mmHg)	83 ± 15	82 ± 17	85 ± 14	83 ± 14	0.83
Pulse rate (beats/minutes)	78 ± 15	79 ± 13	79 ± 16	76 ± 15	0.69
Antihypertensive drug use rates (%)	69.9	54.8	73.1	80.6	0.047
Total protein (g/dL)	7.2 ± 0.4	7.3 ± 0.4	7.1 ± 0.4	7.2 ± 0.3	0.26
Albumin (g/dL)	4.3 ± 0.4	4.3 ± 0.4	4.3 ± 0.4	4.2 ± 0.3	0.92
AST (U/L)	23.3 ± 9.6	24.8 ± 12.8	22.7 ± 5.6	22.4 ± 8.7	0.57
ALT (U/L)	23.6 ± 14.1	25.4 ± 18.9	23.0 ± 11.2	22.4 ± 10.9	0.68
Urea nitrogen (mg/dL) *	15.1 ± 4.3	15.7 ± 4.8	14.1 ± 3.9	15.4 ± 3.9	0.33
Creatinine (mg/dL) *	0.76 ± 0.39	0.83 ± 0.60	0.71 ± 0.23	0.73 ± 0.22	0.47
Uric acid (mg/dL) *	5.0 ± 1.3	5.3 ± 1.4	4.8 ± 1.4	5.0 ± 1.1	0.27
Blood glucose (mg/dL)	116 ± 49	110 ± 23	110 ± 37	126 ± 68	0.30
Serum sodium (mmol/L)	141 ± 3	141 ± 3	143 ± 2	141 ± 3	0.0061
Serum potassium (mmol/L)	3.8 ± 0.5	4.0 ± 0.3	3.3 ± 0.5	4.0 ± 0.4	< 0.0001
Serum chloride (mmol/L)	106 ± 3	104 ± 3	104 ± 3	105 ± 3	0.74
Serum osmolality (mOsm/kg)	288 ± 5	287 ± 3	289 ± 6	288 ± 6	0.23
TTKG	5.1 ± 2.1	4.8 ± 1.7	6.1 ± 2.7	4.7 ± 1.6	0.020
FEK	7.8 ± 4.5	7.5 ± 4.2	8.9 ± 5.9	7.3 ± 3.6	0.34
Median of Plasma renin activity in early morning (ng/mL/h) (IQR)	0.3 (0.2–0.6)	0.8 (0.3–1.8)	0.2 (0.2–0.4)	0.2 (0.2–0.3)	0.0009
Median of Plasma aldosterone concentration in early morning (pg/mL) (IQR)	149 (100–222)	101 (70–165)	245 (206–339)	135 (104–175)	0.0001
Median of Plasma renin activity in the simple standing test (ng/mL/h) (IQR)	0.5 (0.2–1.1)	1.7 (0.8–6.1)	0.3 (0.2–0.6)	0.3 (0.2–0.5)	0.011
Median of Plasma aldosterone concentration in the simple standing test (pg/mL) (IQR)	245 (172–384)	192 (151–280)	296 (206–470)	241 (183–342)	0.11
Localization of adrenal lesions > 10 mm on CT (none/unilateral/bilateral)	35/47/11	17/9/5	1/25/0	17/13/6	< 0.0001
Lateralized ratio of SAVS	3.1 ± 8.4	-	5.8 ± 1.6	1.6 ± 1.4	0.031

Data are presented as mean ± standard deviation. PA, primary aldosteronism; BMI, body mass index; AST, aspartate aminotransferase; ALT, alanine aminotransferase; TTKG, transtubular potassium gradient; FEK, fractional excretion potassium; ARR, aldosterone renin ratio; CT, computed tomography; SAVS, selective adrenal vein sampling; IQR, interquartile range. P values for three-group comparisons using ANOVA are listed.

### Aldosterone/renin ratio in the early morning, simple standing test, and captopril loading test

**Figure 3:**
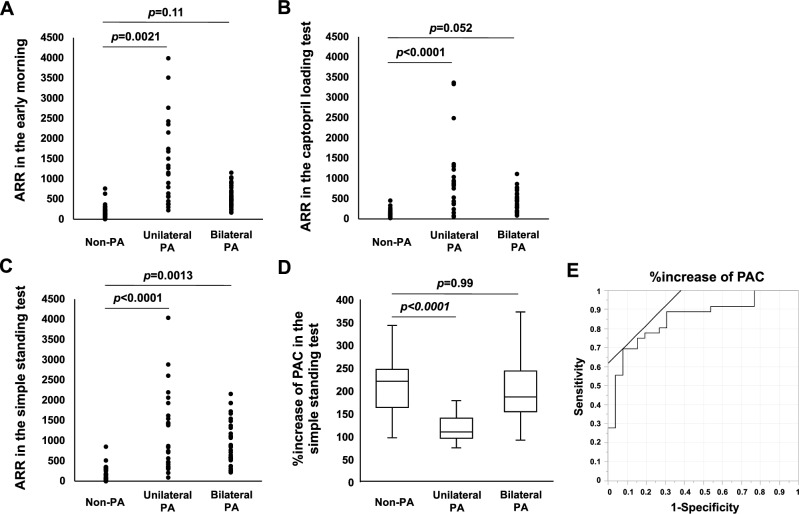
Comparison of each test value in non-PA and unilateral/bilateral PA subjects. (**A**) ARR in the early morning. (**B**) ARR in the captopril loading test. (**C**) ARR in the simple standing test. (**D**) Box-and-whisker diagram showing %increase of PAC for each group. (**E**) ROC curve was developed to calculate cutoff value of %increase of PAC for localization diagnosis of PA subjects.

Figure 3 shows the results of ARR in the early morning, simple standing test, and captopril loading test. ARR in the early morning was 152 (102–240) in non-PA, 1143 (528–2200) in unilateral PA, and 521 (374–765) in bilateral PA subjects (Fig. 3A). The mean ARR in the captopril loading test was 131 (73–196) in non-PA, 850 (281–610) in unilateral PA, and 453 (281–610) in bilateral PA subjects (Fig. 3B). ARR in the early morning and captopril loading test was significantly higher in subjects with unilateral PA compared to non-PA subjects (*p* = 0.0021, *p* < 0.0001, respectively), but there was no significant difference between non-PA and bilateral PA subjects (*p* = 0.11, *p* = 0.052, respectively). The mean ARRs in the simple standing test in unilateral PA (854 (401–1963)) and in bilateral PA subjects (805 (548–1361)) were significantly higher compared to non-PA subjects (129 (61–302)) (*p* < 0.0001, *p* = 0.0013, respectively) (Fig. 3C). The percentage increase of plasma aldosterone concentration (%increase of PAC) after standing was 221 (164–247) in non-PA and 187 (155–244) in bilateral PA subjects, with no difference between them (*p* = 0.99). On the other hand, %increase of PAC in unilateral PA subjects (110 (96–140)) was significantly lower compared to non-PA subjects (*p* = 0.0003) (Fig. 3D). In subjects diagnosed with PA, the cutoff value of %increase of PAC to differentiate between unilateral and bilateral PA was 169 (AUC = 0.853, sensitivity 92.3%, specificity 69.4%, *p* < 0.0001) (Fig. 3E). Table 2 shows the results of nominal logistic regression analysis to assess predictors of PA localization. In this study, %increase of PAC and the presence of unilateral adrenal lesions were factors that inferred a localization diagnosis in PA subjects. Table 3 then evaluates PA subjects divided into groups according to the presence or absence of unilateral adrenal lesions on CT. The results showed that the %increase of PAC was significantly lower in subjects with unilateral PA, regardless of the presence or absence of adrenal lesions (*p* = 0.0003). When %increase of PAC (< 169) was used in combination with unilateral adrenal lesion on CT, the sensitivity and specificity for localization diagnosis in subjects with PA were 96.2% and 100%, respectively (*p* < 0.0001), both of which were higher compared to when %increase of PAC or unilateral adrenal lesions were evaluated alone (Table 3).

**Table 2:** Nominal logistic regression analysis of factors inferring localized diagnosis of PA.

Parameter	Estimated value	Standard error	Wald	*P* value
Age	0.100	0.112	0.805	0.37
Woman	− 0.526	0.713	0.544	0.46
Antihypertensive drug use rates	0.330	1.138	0.084	0.77
%increase of PAC	0.058	0.025	5.320	0.021
Serum potassium	2.639	1.707	2.389	0.12
Unilateral adrenal lesion on CT	− 2.645	1.119	5.586	0.018

PA, primary aldosteronism; 95%CI, 95% confidence interval; PAC, plasma aldosterone concentration; CT, computed tomography.

**Table 3:** Results of simple standing test by unilateral adrenal lesions in unilateral/bilateral PA.

Parameter	Unilateral PA		Bilateral PA		P value
	With unilateral adrenal lesions > 10 mm on CT (n = 25)	Without unilateral adrenal lesions > 10 mm on CT (n = 1)	With unilateral adrenal lesions > 10 mm on CT (n = 13)	Without unilateral adrenal lesions > 10 mm on CT (n = 23)	
Plasma renin activity in early morning (ng/mL/h)	0.2 (0.2–0.4)	0.3	0.2 (0.2–0.4)	0.2 (0.2–0.3)	0.78
Plasma aldosterone concentration in early morning (pg/mL)	254 (218–343)	134	132 (105–167)	141 (97–182)	0.036
ARR in early morning	1155 (563–2250)	447	530 (405–835)	483 (366–705)	0.43
Plasma renin activity in the simple standing test (ng/mL/h)	0.3 (0.2–0.7)	0.30	0.3 (0.2–0.6)	0.3 (0.2–0.5)	0.84
Plasma aldosterone concentration in the simple standing test (pg/mL)	309 (229–487)	144	259 (218–360)	231 (173–345)	0.60
ARR in the simple standing test	870 (389–1997)	480	1175 (447–1608)	764 (578–1099)	0.81
% increase of PAC	110 (94–141)	107	189 (173–279)	187 (130–241)	0.0003

Diagnostic model for PA localization	Positive rate of unilaretal PA	Positive rate of bilateral PA	Sensitivity	Specificity	*P* value
% increase of PAC < 169	92.3	30.6	92.3	69.4	< 0.0001
Unilateral adrenal lesion > 10 mm on CT	96.2	36.1	96.2	63.9	< 0.0001
% increase of PAC < 169, unilateral adrenal lesion > 10 mm on CT	92.3	0	96.2	100	< 0.0001

PA, primary aldosteronism; CT, computed tomography; PAC, plasma aldosterone concentration. P values for four-group comparisons using ANOVA are listed.

### Diagnostic performance of each test in primary aldosteronism

**Figure 4:**
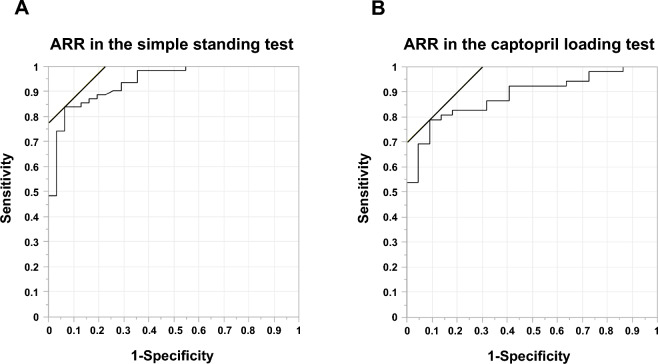
ROC curves were developed to calculate cutoff values for each test to diagnose primary aldosteronism. ROC curve for the ARR in the simple standing test (**A**) and for the ARR in the captopril loading test (**B**).

ROC curves were developed to evaluate the usefulness of each test's ARR for diagnosis of PA (Fig. 4). The results showed that AUC = 0.948, sensitivity = 83.9%, and specificity = 93.5% when ARR 364.0 was used as the cutoff value for the simple standing test (*p* < 0.0001) (Table 4). The diagnostic performance of the captopril loading test in this study was AUC = 0.886, sensitivity = 78.9%, and specificity = 90.1% (*p* < 0.0001) when the cutoff value of ARR after loading was set at 281.4. Applying the early morning ARR 200, a common screening criterion, to the participants in this study, 60 of 62 subjects with PA and 14 of 31 non-PA subjects tested positive (sensitivity 96.7%, specificity 45.2%). When a simple standing test ARR of 364 or higher was used as the criterion and combined with a general screening test, the sensitivity of the test was 98.4% and specificity 45.2%, greater than that of a single test when either ARR in the early morning or the simple standing test was positive.

**Table 4:** Cut-off values for each test for PA diagnosis in this study.

	AUC	Cut-off value	Sensitivity (%)	Specificity (%)	*P* value
ARR in the early morning	0.935	322.5	88.7	90.3	< 0.001
ARR in the captopril loading test	0.885	281.4	78.9	90.1	< 0.001
ARR in the simple standing test	0.938	364.0	83.9	93.5	< 0.001

PA, primary aldosteronism; ARR, aldosterone renin ratio.

## Discussion

In this study, we investigated the diagnostic utility of a simple standing test in patients with primary aldosteronism. The diagnostic performance of the simple standing test for PA was not inferior to that of the captopril loading test. The localization of PA is an important factor in the decision for surgical treatment, and the %increase of PAC in the simple standing test was significantly lower in unilateral PA subjects compared to bilateral PA subjects. In addition, it is likely that the combination of %increase of PAC and CT findings of unilateral adrenal lesions in patients with PA is a more accurate predictor of localization diagnosis. These results demonstrate the usefulness of the simple standing test in the diagnosis of PA, as it does not require medication and can be performed at the same time as general screening tests.

Captopril loading test, furosemide load test, and saline load test are used to diagnose PA^12^. Although these tests are relatively easy to perform, there are concerns about complications such as hypotension or hypertension associated with medications and the long time required to perform the tests. The simple standing test can be performed at the same time as the early morning ARR measurement, which is a screening test for PA, and does not require medication, which is a major advantage. There were no adverse events associated with the simple standing test in the participants of this study, and we think that the test is safe. In the present study, when the cutoff value of the ARR for the simple standing test was set at 364.0, the sensitivity and specificity for diagnosis were no less than those of the captopril loading test. In addition, by evaluating both the early morning ARR and the simple standing test ARR, the sensitivity of the test may be increased by picking up cases in which either is positive.

For patients with unilateral PA, such as aldosterone-producing adenoma (APA) or unilateral adrenal hyperplasia (UAH), laparoscopic adrenalectomy can cure or significantly improve hypertension by improving hyperaldosteronism and hypokalemia^13,14^. Reliable evaluation of PA localization by SAVS is very important in determining treatment strategies, as surgical outcomes are better after localization by SAVS than when localization is diagnosed by CT or MRI alone^15,16^. Clinical prediction tools that infer the diagnosis of PA localization from test results such as age, gender, unilateral adrenal lesion, and saline tolerance test have been studied, but results may differ among populations, and new clinical prediction tools that do not require SAVS should be developed^17^. In this study, we evaluated %increase of PAC in simple standing test and found that it was significantly smaller in unilateral PA subjects compared to bilateral PA subjects. Previous studies showed that %increase of PAC in simple standing studies of aldosterone-producing adenomas were 135 ± 42%^10^. Recently, other centers have also reported that plasma aldosterone concentrations after 4 h of standing load are useful for detecting PA by APA in patients with a 28% decrease in plasma aldosterone concentrations^11^. These results were similar to those of the present study. Several studies have been reported evaluating differences in the tendency to secrete aldosterone with postural stimulation and the diagnosis of PA localization^18–20^, and it is possible that bilateral PA is hyperresponsive to postural stimulation compared to unilateral PA. In the present study, the %increase of PAC in the simple standing test was found to be useful in predicting the diagnosis of PA localization, with higher sensitivity and specificity compared to previous reports. The high diagnostic accuracy of this study may be due to the inclusion of more participants with bilateral PA in the analysis and the absence of participants with angiotensin II reactive adenoma, which causes hyperresponsiveness in the simple standing test^21^. Alternatively, the postural stimulation time may have been different from previous reports. A multicenter clinical study including PA patients with characteristics of angiotensin II- and ACTH-responsive adenomas would be desirable to infer generalized criteria for the simple standing test.

Interestingly, among the subjects diagnosed with unilateral PA by SAVS, the %increase of PAC in one subject who did not have unilateral adrenal lesions on CT was 107%, which was similar to those in other subjects diagnosed with unilateral PA. Among the subjects diagnosed with bilateral PA by SAVS, 13 subjects with unilateral adrenal lesions on CT had an average %increase of PAC of 224 ± 70%. Although unilateral adrenal lesions on CT alone may be difficult to localize, the combination of unilateral adrenal lesions on CT and %increase of PAC on simple standing test could be used to predict lesion localization in patients with PA, which was similar to previous reports^22^. It is noted that the results of our work were not validated on any cohort of patients, although we independently confirmed the results of the previous work. Therefore, we think that the result in this study is an indicator that could be used in a limited cohort of patients and that further research is warranted to generalize this indicator.

This study has several limitations. First, this is a single-center, backward-looking observational study. The participants who underwent the simple stance test at our institution were those suspected of having PA based on some symptoms or test results, which may have intervened in the selection bias. In addition, non-PA subjects did not undergo SAVS, which may include patients with PA who had false-negative captopril loading tests and simple standing tests. Second, this study did not include any patients with an angiotensin II-responsive adenomas. We think that the sensitivity and specificity of the test would be reduced in patients including those with angiotensin II-responsive adenomas, because it is possible that the increased autonomous aldosterone secretion reduces the responsiveness of aldosterone secretion in simple standing test. Since the percentage of angiotensin II- and/or ACTH-responsive adenomas may vary by region and patient background among various centers, we think that it would be necessary to analyze a large number of patients from different geographical areas to establish the usefulness of simple standing test in clinical practice. Third, this study excluded other adrenal diseases such as adrenocortical Cushing's syndrome and pheochromocytoma. Comorbidities may lead to different results than in the present study. Fourth, plasma aldosterone levels were measured by the RIA method in this study, since it has become standard in Japan since 2022 that plasma aldosterone levels are measured by the CLEIA method. Therefore, the results may differ due to differences in testing methods. And the higher rate of antihypertensive drug at admission in unilateral/bilateral PA subjects compared to non-PA subjects may have influenced the results^23^. In addition, the saline stress test and the furosemide standing stress test, which are PA screening tests as well as the captopril stress test, could not be compared with the results of the simple standing test due to the low rate of performance at our hospital. Prospective studies are desirable for comparison with these tests. Finally, this study was conducted in inpatients, and the testing time was constant. Assuming an ambulatory care setting, similar results may not be obtained with a simple standing test if the timing of the test varied depending on the time of the visit. Future studies using ARR at any time and simple standing tests in the outpatient setting are desirable.

In conclusion, this study demonstrates that in the practice of primary aldosteronism, the simple standing test has a test specificity comparable to that of the captopril loading test and that its combination with the early morning ARR enhances test sensitivity. In addition, the data in this study suggest that the %increase of PAC in the simple standing test is a possible aid in localization diagnosis. In particular, it is likely that the combination of unilateral adrenal lesions on CT and a low %increase of PAC is more useful for localization diagnosis in patients with PA. The simple standing test is a very simple test that does not require medication and can be performed by primary care physicians and non-specialized medical facilities. Therefore, the simple standing test may be considered as a screening test for PA. Future studies in various areas are encouraged to examine the usefulness of the simple standing test as a clinical predictive tool for PA.

## Data Availability

All data generated or analysed during this study are included in this published article.
